# Involvement of three chemosensory proteins in perception of host plant volatiles in the tea green leafhopper, *Empoasca onukii*

**DOI:** 10.3389/fphys.2022.1068543

**Published:** 2023-01-04

**Authors:** Liwen Zhang, Mingxian Zhao, Feiruoran Aikeremu, Huoshui Huang, Minsheng You, Qian Zhao

**Affiliations:** ^1^ State Key Laboratory for Ecological Pest Control of Fujian/Taiwan Crops and College of Life Science, Fujian Agriculture and Forestry University, Fuzhou, China; ^2^ Key Laboratory of Integrated Pest Management for Fujian-Taiwan Crops, Ministry of Agriculture and Rural Affairs, Fuzhou, China; ^3^ International Joint Research Laboratory of Ecological Pest Control, Ministry of Education, Fujian Agriculture and Forestry University, Fuzhou, China; ^4^ Comprehensive Technology Service Center of Quanzhou Customs, Quanzhou, China

**Keywords:** chemosensory proteins, *Empoasca onukii*, insect chemoreception, plant volatiles, benzaldehyde

## Abstract

Chemosensory proteins (CSPs) can bind and transport odorant molecules, which are believed to be involved in insect chemoreception. Here, we investigated three CSPs in perception of volatiles in *Empoasca onukii*. Expression profiles showed that although *EonuCSP4*, *EonuCSP 6-1* and *EonuCSP6-2* were ubiquitously expressed in heads, legs, thoraxes and abdomen, they were all highly expressed in the antennae of *E. onukii*. Further, fluorescence competitive binding assays revealed that *EonuCSP4* and 6-1 had binding affinities for three plant volatiles, suggesting their possible involvement in the chemosensory process. Among them, *EonuCSP6-1* showed relatively high binding affinities for benzaldehyde. Behavioral assays revealed that the adults of *E. onukii* showed a significant preference for two compounds including benzaldehyde. The predicted three-dimensional (3D) structures of these 3 CSP have the typical six α-helices, which form the hydrophobic ligand-binding pocket. We therefore suggest that *Eoun6-1* might be involved in the chemoreception of the host-related volatiles for *E. onukii*. Our data may provide a chance of finding a suitable antagonist of alternative control strategies which block the perception of chemosensory signals in pest, preventing the food- orientation behaviors.

## Introduction

Molecular sensing of chemicals is important for locating food, searching mating partners, selecting oviposition sites, socializing among groups, and avoiding predators of insects (Bruce et al., 2005; Ebrahim et al., 2015; Pelosi et al., 2018). The communication of the insect chemosensory system is characterized by stability, sensitivity and specificity (Carey et al., 2010; Mao et al., 2010; Leal 2013). Multiple olfactory proteins involved in the odorants perception were identified (Hua et al., 2021; Zhan et al., 2021; Zhang et al., 2021). Firstly, hydrophobic odorants enter the insects through the olfactory pores of cuticular chemosensilla (Stocker 1994; Wang et al., 2020). Secondly, odorant binding proteins (OBPs) and chemosensory proteins (CSPs) bind to hydrophobic odorants and transport them to the olfactory sensory neurons (OSNs) (Vogt and Riddiford 1981; Zhang et al., 2018; Zhang et al., 2021). Then, odorant receptors (ORs) as odorant-gated ion channels play roles in insect olfaction. They are formed by a heteromultimeric complex of the odorant receptor co-receptor (Orco) and a ligand-selective Or, which are activated when the odorant binds to the binding site (Laughlin et al., 2008; Sato et al., 2008; Wicher et al., 2008; Stengl and Funk 2013; Butterwick et al., 2018; Del Mármol et al., 2021) and convert the chemical signals into nerve impulses (Ha and Smith 2009). Next, odorants are degraded by odorant degrading enzymes (ODEs) (Ishida and Leal 2005; Wang M. M. et al., 2021). Subsequently, nerve impulses are transmitted to the central nervous system (CNS) to guide the insects’ behaviors (Leal et al., 2005).

CSPs are small water-soluble proteins and are major binding proteins in insects with small molecular weight of ∼12 kDa. CSPs possess a conserved cysteine CSP motif (C1-X_6-8_-C2-X_16-21_-C3-X_2_-C4) that form two disulfide bridges (C1-X_6-8_-C2, C3-X_2_-C4) (Zhou et al., 2006). The disulfide bonds in the CSP motif are inter-helical with two small loops, forming a rigid hydrophobic pocket involved in ligand binding (Lartigue et al., 2002). This folding conformation are different from those of insect OBPs (Tegoni et al., 2004). Some CSPs function in chemosensory signal transduction and solubilization of pheromone components (Zhan et al., 2021; Zhang et al., 2021). Other CSPs are involved in insect physiological processes and behavior, such as moulting, tissue formation, regeneration, reproduction and resistance (Li et al., 2015; Zhu et al., 2016; Pelosi et al., 2018). *BodoCSP1* is involved in host plant volatiles perception in *Bradysia odoriphaga* (Zhang et al., 2021). *CforCSP1, 5* and *6* are involved in the chemical communication between *Cylas formicarius* and host plant volatiles (Hua et al., 2021). Besides the chemosensory functions, CSPs are found to have various physiological functions. In *Locusta migratoria*, CSPs are involved in the physiological transition from solitary to the gregarious phase (Guo et al., 2011). *CSP5* in *Apis mellifera* functions in embryo development (Maleszka et al., 2007). In *Solenopsis invicta, CSP9* is responsible for cuticle development (Cheng et al., 2015). In *Periplaneta Americana, CSP10* participates in leg regeneration (Kitabayashi et al., 1998). *BmorCSPs* of *Bombyx mori* are associated with insect resistance to insecticide abamectin (B1a and B1b avermectins) (Xuan et al., 2015).

The tea green leafhopper, *Empoasca onukii* (*Hemiptera*: *Cicadellidae*) is a serious pest of tea plants in East Asia. The nymphs and adults suck the sap from the fresh buds while female adults lay eggs in tender plant shoots, which leads to early symptoms of chlorosis and leaf curling, followed by browning, shriveling and necrosis that ultimately destroy the whole leaf (Jin et al., 2012). Outbreaks of *E. onukii* lead to significantly reduction of tea yields both in summer and autumn, two periods of tea harvest, with economic losses up to 15%–50% in China (Chen et al., 2019) and 33% in Japan (Zhang et al., 2019). For now, chemical insecticides remains the main control strategy for its management. However, the development of insecticide resistance/tolerance in the tea green hopper makes its control difficult. Meanwhile, the excessive use of insecticides damages the ecological environment and causes the residue problems of tea. Therefore, it is urgent to develop the safe strategies to control this pest. Understanding the mechanism and signals that are involved in the odorant reception of *E. onukii* may provide the clues of control methods, which enhance the tea safety.

Twenty-six species-expanded CSP genes have been recently identified in *E. onukii* based on genome data (Zhao et al., 2022). However, there is no information about the CSPs that can bind to the volatiles from tea, which can be further used to develop the alternative control strategies against *E. onukii*.

In the current study, we focused on analyzing the CSPs distributing in clusters on Chromosome 1. Results showed that there are 7 CSPs distributing in clusters on chromosome 1. However, only 3 CSPs were successfully expressed in the expression vector. Bases on phylogenetic analysis with aphid, plant bug, and plant hoppers, these 3 CSPs belonged to aphid CSP4 and aphid CSP6 cluster respectively. We named them as *EonuCSP4*, *EonuCSP6-1*, and *EonuCSP6-2*. To identify the roles of these CSPs, we analyzed their expression patterns. Besides, binding properties of the 3 CSPs were investigated by fluorescence binding assays. In addition, we use a Y-tube olfactometer to detect the behavioral responses of *E. onukii* to volatiles. These results provide clues for better understanding the chemosensory mechanisms of *E. onukii*, which lead to the novel way of pest control strategies.

## Materials and methods

### Sample collection, total RNA extraction and cDNA synthesis

*E. onukii* adult samples were collected in Fuzhou, Fijian province, southeastern China in May 2020 (on the tea cultivar of Fudingdabai), and maintained on tea plants in lab. The insectarium environment was set at 25°C ± 1°C and 60 ± 5% relative humidity (RH) with a photoperiod (light: dark = 14:10).

Total RNA was extracted using Eastep^®^ Super Kit (Promega, Beijing, China) according to the protocol. The concentration of RNA was assessed by NanoDrop 2000 spectrophotometry (NanoDrop, DE, USA) by measuring the OD at 260 nm. Then, the cDNAs were synthesized using FastKing gDNA Dispelling RT SuperMix (Tiangen, Beijing, China) according to the protocol.

### Phylogenetic analysis

CSP sequences from plant bugs, aphid and plant hoppers were from previous study (Wang Q. et al., 2019). Sequences alignments were conducted with the MUSCLE alignment program (http://www.drive5.com/muscle/manual/). We used Neighbor-Joining algorithm (NJ) to construct unrooted phylogenies of these sequences with MEGA7 (Kumar et al., 2016). Bootstrap values were calculated with 1000 replicates; bootstrap values < 50% were deleted from the branches.

### 3D modeling

We used the strategies to predict the 3D modeling of the CSPs, which was described before (Zhang et al., 2020). By homology searching, the CSPs that we identified have > 40% homology with the CSP templates in the Protein Data Bank (http://www/rcsb.org/pdb). Based on the high sequence similarity (Supplementary Table S2) with the 3 *E. onukii* CSPs, NMR solution structure of CSPsg4 (PDB ID: 2gvs.1.A) was used as the template to build the 3D structures of the 3 CSPs using the online program SWISS MODEL. Information of the template was shown in Supplementary Table S2. The final 3D model was assessed using Verification Server (http://services.mbi.ucla.edu/SAVES/).

### Expression patterns analysis

Body parts were collected from both adult males and females including head, thorax, abdomen, leg. Different parts were dissected from 1 to 5 days old adults (300 males and 300 females). Five biological replicates were prepared. Total RNA was extracted following the protocols above (section of Total RNA extraction and cDNA synthesis). We performed the qPCR to analyze the expression patterns of the 3 CSP genes (primers are listed in Supplementary Table S1). qPCR was performed on ABI Prism 7500 Fast Detection System (Applied Biosystems, Carlsbad, CA, United States). Each reaction contained 10 μl of 2 × GoTaq qPCR Master Mix, 0.4 μl of each primer (10 μM), 7 μl of nuclease-free water, 0.2 μl of CXR References Dye and 2 μl of sample cDNA (500 ng μl^−1^). The thermocycler program had an initial 95 denaturation step followed by 40 cycles consisting of a 10 s denaturation at 95, a 40 s annealing at 60, and a 30 s extension step at 72. The relative expression levels of BodoCSP1 were analyzed using the 2^−ΔΔCT^ method (Li et al., 2021). β-actin gene was used as a control to normalize target gene expression and correct for sample-to-sample variation.

### Bacterial expression and purification of recombinant CSP proteins

Primers designed for constructs were shown in Supplementary Table S1. Purified PCR products were ligated into the expression vector pET32a (+) with TRX-6 His tag (∼17 kDa) and the resulting construct were transformed into *Escherichia coli* BL21 (DE3) competent cells. These recombinant plasmid CSPs were firstly confirmed by sequencing. Bacteria transformed with recombinant plasmids were cultured in 1000 ml Luria−Bertani (LB) medium containing 100 μg/ml of ampicillin. Then the recombinant protein was induced at 16°C for 24 h with 1 mM isopropyl β-d-1-thiogalactopyranoside (IPTG) when OD600 reached 0.4–0.6. We performed the centrifugation to collect the cells at 8000g for 5 min at 4°C and then sonicated in ice. The recombinant protein was further purified by Ni-NTA resin (GE). The poly-histidine tag was not removed following studies before (Terpe. 2003; Li et al., 2016; Tian and Zhang. 2016; Cui et al., 2018; Zhang et al., 2020). Purified protein was verified by sodium dodecyl sulfate polyacrylamide gel electrophoresis (SDS-PAGE). Finally, purified protein was dialyzed with Buffer B. The molecular weights of recombinant EonuCSP proteins are consist with the predicted molecular weight of EonuCSPs (predicted at https://web.expasy.org/protparam/) plus a TRX-6 His tag (∼ 17 kDa).

### Competitive fluorescence binding assays

Based on the results of previous studies (Zhao et al., 2002; Zhang et al., 2012; Zhao et al., 2014; Cai et al., 2017; Xu et al., 2017), we chose 15 typical volatile components as ligands for the fluorescence competitive binding assays (Table 1). These volatile components are the volatile components in tea (Cai et al., 2017) with purity > 98% (Table 1). N-phenyl-1-naphthylamine (1-NPN) was used as the fluorescent probe. The excitation wavelength in the fluorescence spectrometer was set to 337 nm, which was the results of an optimization that we achieved in lab. The scanning wavelength was 420–600 nm.

**TABLE 1:** Binding affinities of all tested ligands to the 3 CSPs in *E. onukii.*

Ligands	Source	CAS number	Purity (%)	EonuCSP4		EonuCSP 6-1		EonuCSP6-2	
				IC_50_ (*μ*M)	*K*_*i*_ (*μ*M)	IC_50_ (*μ*M)	*K*_*i*_ (*μ*M)	IC_50_ (*μ*M)	K_i_ (*μ*M)
Methyl Salicylate	TCI	119-36-8	>99%	U.d.	U.d.	U.d.	U.d.	U.d.	U.d.
Ocimene	MACKLIN	13877-91-3	≥90%	21.96	18.93	U.d.	U.d.	U.d.	U.d.
Cis-3-Hexenyl n-valerate	TCI	35852-46-1	>98%	U.d.	U.d.	U.d.	U.d.	U.d.	U.d.
β-ionone	MACKLIN	79-77-6	95%	U.d.	U.d.	U.d.	U.d.	U.d.	U.d.
Ethyl Benzoate	J&K	93-89-0	>99%	U.d.	U.d.	U.d.	U.d.	U.d.	U.d.
Benzaldehyde	MERCK	100-52-7	99%	U.d.	U.d.	21.32	18.54	U.d.	U.d.
Cis-3-Hexrnyl Butyrate	MACKLIN	16497-36-4	≥98%	U.d.	U.d.	U.d.	U.d.	U.d.	U.d.
(-)-Linalool	MACKLIN	126-91-0	≥95%	U.d.	U.d.	U.d.	U.d.	U.d.	U.d.
Ethyl Decanoate	TCI	110-38-3	>98%	U.d.	U.d.	U.d.	U.d.	U.d.	U.d.
Cis-3-Hexenyl Hexanoate	MACKLIN	31501-11-8	98%	U.d.	U.d.	U.d.	U.d.	U.d.	U.d.
Cis-3-Hexen-1-ol	ALADDIN	928-96-1	98%	U.d.	U.d.	U.d.	U.d.	U.d.	U.d.
Limonene	MACKLIN	5989-27-5	>99%	U.d.	U.d.	U.d.	U.d.	U.d.	U.d.
Farnesene	ALADDIN	502-64-4	98%	20.17	17.85	U.d.	U.d.	U.d.	U.d.
trans-β-Farnesene	ALADDIN	18794-84-8	98%	U.d.	U.d.	U.d.	U.d.	U.d.	U.d.
3-Carene	TCI	13466-78-9	90%	U.d.	U.d.	U.d.	U.d.	U.d.	U.d.

“U.d.” means that the *K*_i_ exceeded 20 μM. Low *K*_i_ values mean high binding affinity for protein and the test volatiles. Purity (%) showed the ligand concentration range.

The tested chemicals were dissolved in methanol in preparation for 1 mM stock solution. To measure the affinity of 1-NPN to the CSP proteins, 2 μM solution of purified protein in buffer B was titrated with aliquots of 1 mM 1-NPN dissolved in methanol to final concentrations ranging from 2 to 20 μM. Then the affinities of ligands were tested by competitive binding assays through titrating the chemical competitor from 2 to 20 μM into the 1-NPN and EonuCSPs mixed solution (both at 2 μM). We determined the binding constants of 1-NPN by Scatchard formula. The dissociation constant (*K*_d_) for binding between CSPs and 1-NPN was determined with the Scatchard linear regressive equation in the software GraphPad Prism 5.0 (GraphPad Software Inc., La Jolla, CA). The binding affinities of the competitors were evaluated from the corresponding IC_50_ according to the equation: *K*_i_ = IC_50_/(1 + [1-NPN]/K_1-NPN_). [1 – NPN] is the free concentration of 1 – NPN, while K_1-NPN_ is the dissociation constant of the protein/1-NPN complex (Zhang et al., 2021). Tested compounds with *K*_i_ < 20 μM shows relatively high binding affinities to EonuCSPs.

### Y-tube olfactometer assay

We use a Y-tube olfactometer (1.5 cm in diameter, arms with 8 cm length, and a stem with 18 cm length) to detect the behavioral response of *E. onukii* to volatiles. The incoming air to the tube was firstly filtered using active carbon and then humidified with the ultrapure water. The air through the arms was blown at a constant flow (300 ML/min). A total of 20 μL tested volatile oil (10 μg/μL in methanol (HPLC)) pipetted onto 1 cm diameter filter paper. This filter paper was placed in the chamber, which connected to one arm of the Y-tube olfactometer. As control, the same filter paper treated with 20 μL of methanol was placed in the chamber connected to another arm of the Y-tube. This experiment was performed in the dark room, which employed a light-emitting diode as the light source. Each adult was introduced into the middle of the stem of the Y-tube and the individuals that made a choice within 8 min were recorded. Individuals moving toward the odorant source for half of the arm distance and stayed for 1 minute were recorded as odorant choice. For each volatile component, 90 adult were used. After 10 insects were tested, the Y-tube olfactometer was washed with 75% ethanol and air-dried, and tested volatiles were placed in another arm for the subsequent tests. Odor resource are shown in Table 1.

## Result

### Sequence and homology analysis

**FIGURE 1:**
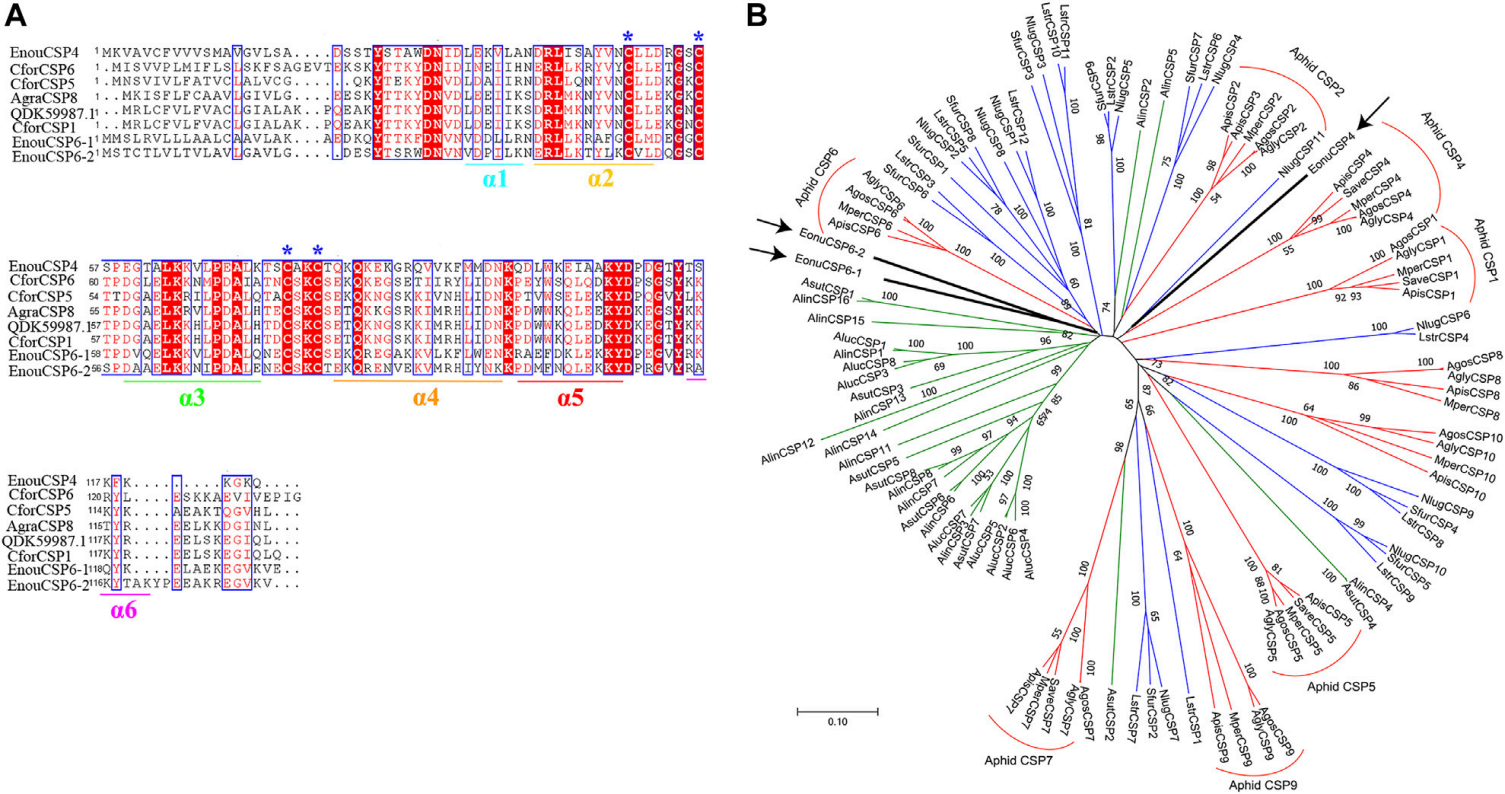
Homology analysis and phylogenetic relationship of EonuCSPs with CSPs from other insects. **(A)**. Secondary structure of *E. onukii* chemosensory proteins (*EonuCSP4, 6-1*, and *6-2*). α -helices are displayed as straight. Identical residues are highlighted in white letters with a red background. Residues with similar physicochemical properties are shown in red letters with a blue frame. The conserved cysteines are labeled with asterisk. **(B)**. Phylogenetic relationships of the CSPs. Species includes hoppers (*N. lugens* (Nlug)*, S. furcifera* (Sfur), and *L. striatellus* (Lstr)], aphids (*A. pisum* (Apis)*, M. persicae* (Mper)*, A. gossypii* (Agos)*, A. glycines* (Agly)*,* and *S. avenae* (Save))*,* and plant bugs (*N. lugens* (Alug)*, L. striatellus* (Lstr)*,* and *S. furcifera* (Sfur)). *E. onukii* CSPs was marked with arrowhead. CSPs are classified into 8 subfamilies in Aphid based on previous study (Wang Q. et al., 2019), which were marked in red. The 3 CSPs belonged to aphid CSP4 and aphid CSP6 cluster respectively. Bootstrap values were calculated with 1000 replicates; bootstrap values < 50% were deleted from the branches.

The full length cDNAs encoding *EonuCSP4*, *6-1*, and *6-2* were amplified (Supplementary Figure S1), cloned and further verified by sequencing. The gene sequences were submitted to GenBank with accession number MF509603.1, MF509616.1 and MF509617.1. According to the genome annotation of *E. onukii* (Zhao et al., 2022), these 3 CSPs are located on Chromosome 1, with *EonuCSP6-1* and *6-2* tandemly distributed (Supplementary Figure S2). Sequence analysis showed that their ORF sequences are 372 bp, 396 bp, and 399 bp respectively (Supplementary Table S3). Meanwhile, the *EonuCSP 4, 6-1* and *6-2* proteins were predicted to contain the signal peptides of 18 amino acides at their N-terminus. Furthermore, the alignments of these cloned CSPs were done with homologues from other insects (Figure 1A). As the results showed, *EonuCSP4, 6-1*, and *6-2* were found to contain typical characteristics including 4 conserved cysteine residues with the following pattern: C1-X6-8-C2-X16-21-C3-X2-C4 (Figure 1A).

Moreover, we searched the CSPs in other Hemiptera species including aphid (*Acyrthosiphon pisum* (Api), *Myzus persicae* (Mpe), *Aphis gossypii* (Ago), *Aphis glycines* (Agl) and *Sitobion avenae* (Sav)), plant bugs (*Adelphocoris lineolatus* (Alin), *Adelphocoris suturalis* (Asu), *Apolygus lucorum* (Alu)), and plant hoppers [*Empoasca onukii* (Eon), *Laodelphax striatellus* (Lst), *Nilaparvata lugens* (Nlu) and *Sogatella furcifera* (Sfu)]. Based on the results, EonuCSPs are clearly clustered together to form 2 homologous subgroups named CSP4 and CSP6 supported by high bootstrap values (Figure 1B).

### 3D model of the 3 EonuCSP

**FIGURE 2:**
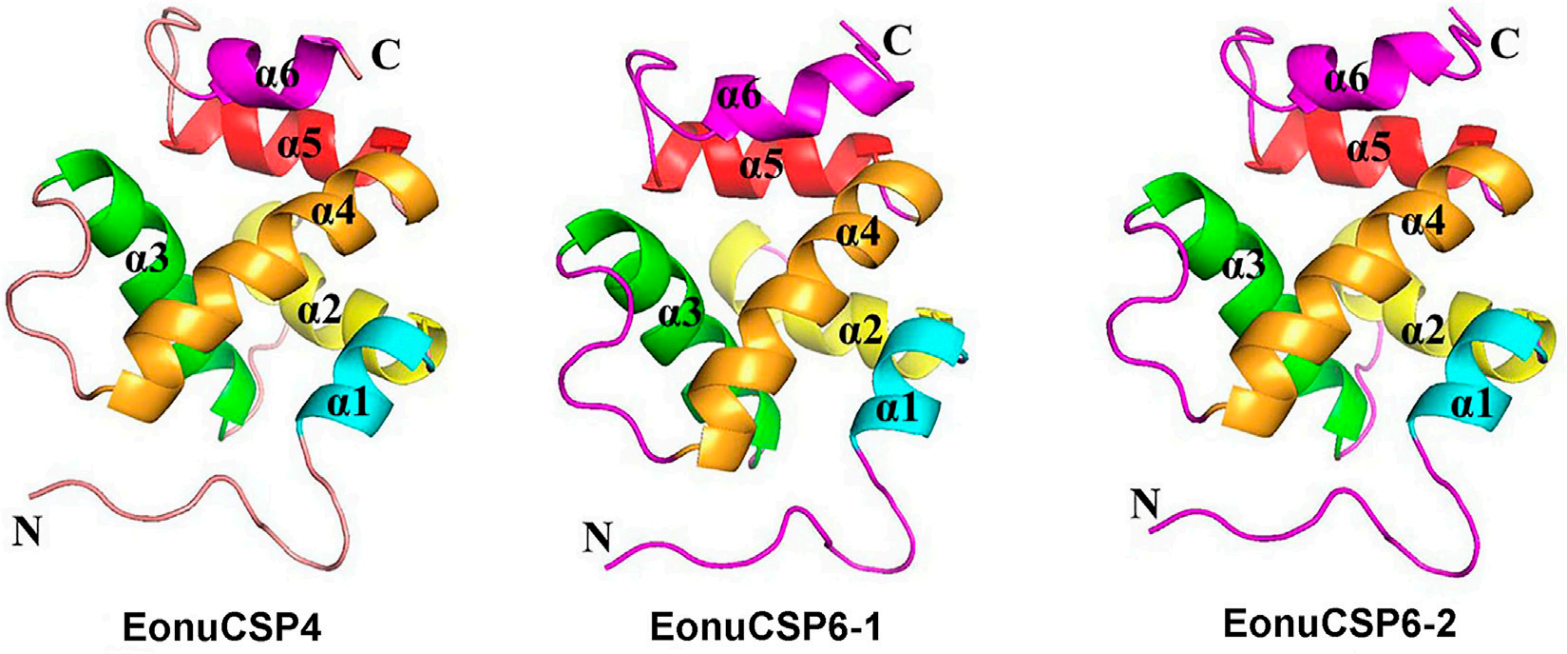
Three-dimensional structure of EonuCSPs. N-terminus, C-terminus, and helices are labeled. The six α-helices are labeled as α1- α6.

The 3D structurally determined CSP, chemosensory protein CSP-sg4 (PBD ID: 2gvs.1.A) was found to share sequence similarities of more than 45% with the 3 CSPs (Supplementary Table S2). Thus, we selected this structurally determined CSP as template to build the 3D structural model of *EonuCSP4, 6-1*, and *6-2* using SWISS-MODEL (Figure 2). The results or Ramachandran plot showed that 87.9%, 85.1%, and 87.2% of the residues were in preferred regions, all the residues (100%) were in the allowed region (Supplementary Figure S3), suggesting that the predicted models of the 3 CSPs are generally reliable. The predicted 3D structures of these 3 CSPs consist of six α-helices (Figures 1A, 2).

### Expression profile of *EonuCSP4, 6-1* and *6-2*

**FIGURE 3:**
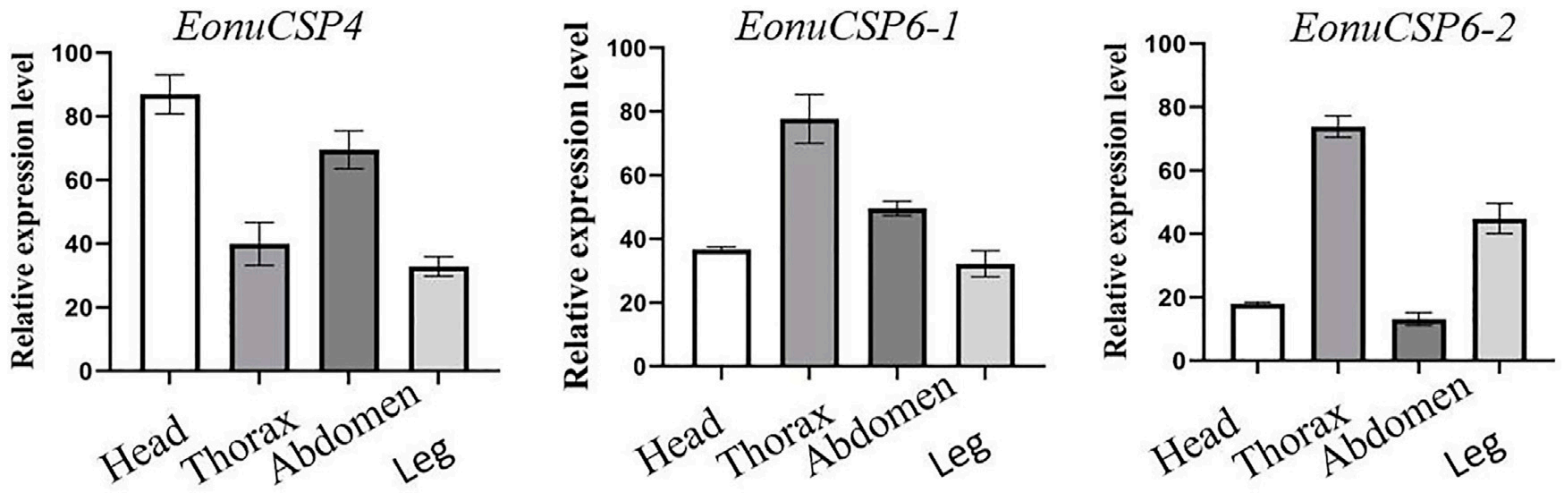
Expression profiles of the 3 CSPs in *E. onukii.* Transcript levels of the CSPs were normalized byβ-actin.

Based on the previous assessment, *EonuCSP4*, *6-1*, and *6-2* were predominantly expressed in antennae (Zhao et al., 2017). For more accurate estimation of the expression profiles, we analyzed the expression patterns of the CSPs in multiple body parts. Results showed that none of the 3 CSP genes were expressed in a specific body part (Figure 3). *EonuCSP4* was expressed higher in the head and abdomen (*p* < 0.05); while *EonuCSP6-1* and *EonuCSP6-2* exhibited a higher expression in thorax than in other body parts (*p* < 0.05; Figure 3).

### Bacterial expression and purification of the 3 EonuCSP

**FIGURE 4:**
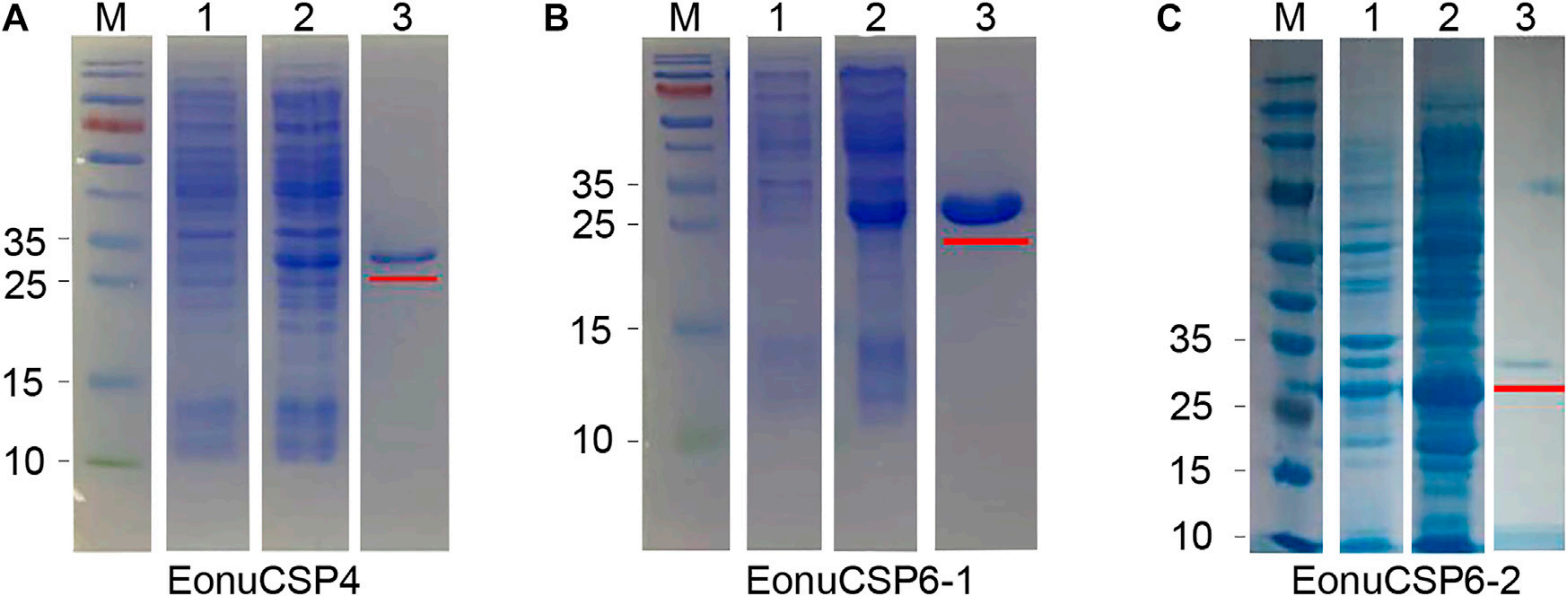
Recombinant protein analyzed by SDS-PAGE. **(A)**. Expression and purification of recombinant protein *EonuCSP4*; **(B)**. Expression and purification of recombinant protein *EonuCSP6-1*; **(C)**. Expression and purification of recombinant protein *EonuCSP6-2*. Lane M: molecular marker. Lane 1: cell pellet before induction with IPTG. Lane 2: cell pellet after induction with IPTG. Lane 3: purified protein, which was also marked in red lines.

The high expression of the 3 CSPs in the antennae suggested that they were potentially involved in peripheral olfactory reception for *E. onukii*. In order to screen the putative ligands for these CSPs, we first expressed the 3 CSPs in a bacterial system. pET-32a (+)/EonuCSPs were successfully induced and expressed in BL21 (DE3) cells. All these 3 EonuCSPs were mainly present in the supernatant. Subsequently, expression and purification of EonuCSP4, *EonuCSP 6-1* and *EonuCSP6-2* recombinant proteins containing His-Tag were assessed by SDS-PAGE with the molecular weight of ∼30.61 kDa, ∼32.06 kDa, and ∼32.12 kDa respectively (Figure 4).

### Fluorescence binding assay

**FIGURE 5:**
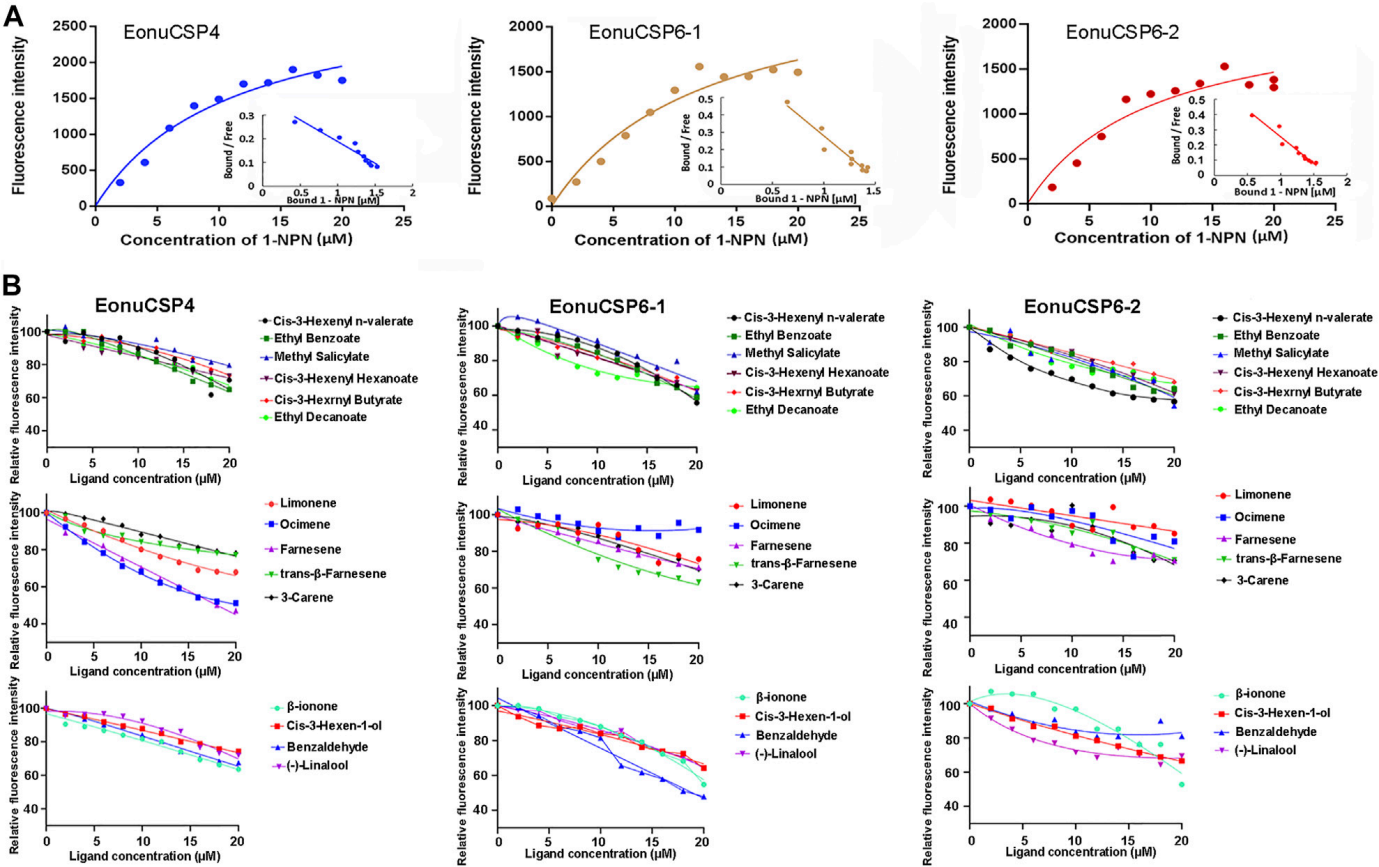
Fluorescence competitive ligand-binding assays of EonuCSPs. **(A)**. Binding curve and the Scatchard plot of N-phenyl-1-naphthylamine (1-NPN) to *EonuCSP4, 6-1*, and *6-2* respectively. **(B)**. Competitive binding curves of *EonuCSP4, 6-1*, and *6-2* with various ligands including ester substances (Cis-3-Hexenyl n-valerate, Ethyl Benzoate, Methyl Salicylate, Cis-3-Hexenyl Hexanoate, Cis-3-Hexrnyl Butyrate, and Ethyl Decanoate), alkene substances (Limonene, Ocimene, Farnesene, trans-β-Farnesene, and 3-Carene), and aldehydes, ketones and alcohols [(-)-Linalool, Cis-3-Hexen-1-ol, β-ionone, and Benzaldehyde]. Dissociation constants (*K*_i_) of all examined ligands were evaluated. Low *K*_i_ values mean high binding affinity for protein and the plant volatiles.

For the ligand binding assay, the binding affinity of the fluorescent probe 1-NPN with the 3 purified CSP proteins were firstly measured (Figure 5A). Results revealed that the 3 purified CSP proteins (*EonuCSP 4, 6-1* and *6-2*) were capable of binding 1-NPN with the dissociation constants (*K*_d_) were 11.02, 12.52 and 10.22 μM respectively. Then, the fluorescence competitive binding assay was performed to determine the binding affinities of *EonuCSP4, 6-1* and *6-2* (Figure 5B). Results revealed that different CSP protein displayed various binding spectrum (Table 1; Figure 5B). And of the 15 tested compounds, each CSP showed a relatively narrow binding spectrum (Table 1; Figure 5B). For *EonuCSP4*, the binding test results indicated that this CSP showed much higher affinity to farnesene (*K*_i_ = 17.85) and ocimene (*K*_i_ = 18.93); whereas *EonuCSP6-1* showed binding to benzaldehyde (*K*_i_ = 18.54). However, *EonuCSP6-2* showed little binding to all the tested compounds, with *K*_i_ >20.

### Behavioral trials

**FIGURE 6:**
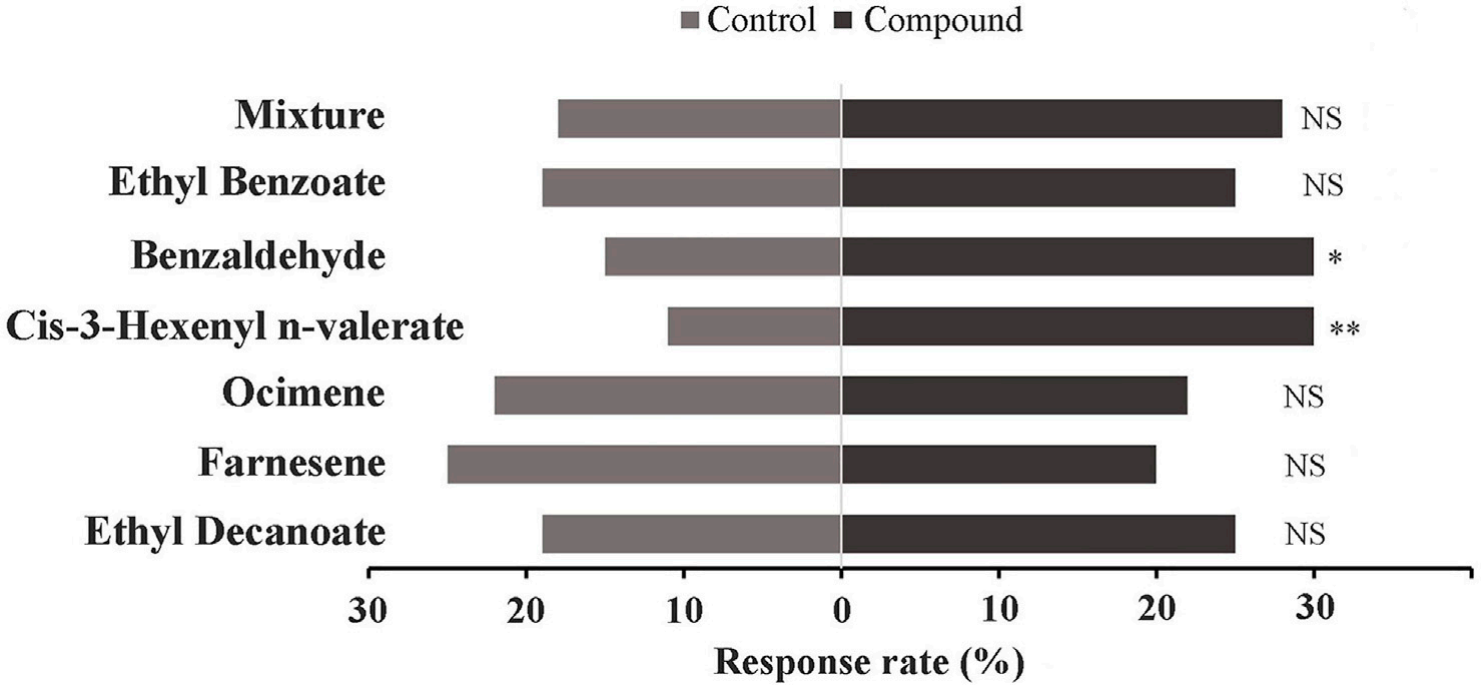
Response of *E. onukii* to 10 μg/μL compounds. Asterisks indicate significant differences; *p < 0.05, and **p < 0.01. NS indicates no significant difference (χ^2^ test). Two compounds, Benzaldehyde (chi-square: 5; *p* value: 0.026) and Cis-3-Hexenyl n-valerate (chi-square: 8.8; *p* value: 0.003), showed significant differences in this assay.

The behavioral responses of *E. onukii* adults to the tested compounds were investigated in a Y-tube olfactometer. Results showed that the adults of *E. onukii* displayed a significant preference for benzaldehyde and cis-3-hexenyl n-valerate (Figure 6), suggesting that other volatiles had no significant effect on the behavior of these insects at concentration of 10 μg/μl.

## Discussion

CSPs of insects could recognize, bind, and transport the chemicals to odorant receptors, which play vital roles in chemoreception (Leal 2013; Pelosi et al., 2018; Zhang et al., 2021). Insect CSPs are also widely expressed in various tissues, which are proved to be involved in multiple physiological processes (Iovinella et al., 2013; Zhao et al., 2017). In this study, 3 CSPs of *E. onukii* were cloned from *E. onukii*, and the respective proteins they encoded were determined to have four typical conserved cysteine spacing motif, which showed a relative high identity with other insect CSPs (Zhang et al., 2020; Hua et al., 2021; Zhan et al., 2021). We investigated the biological binding characteristics of the 3 *E. onukii* CSPs to various plant volatiles. In addition, the 3 CSPs were functionally characterized using behavior bioassays to investigate the involvement of the examined CSPs in plant–volatile detection and plant host orientation behavior.

CSPs of insects exhibit broad expression profiles in various tissues (Wang Z. et al., 2021). qRT-PCR results also showed that the expression of *EonuCSP 4*, *6-1* and *6-2* were expressed in various body parts. Based on the transcriptomes of *E. onukii*, these three CSPs were also expressed in both antennae and body (Zhao et al., 2017). These broad and diverse expression profiles of *EonuCSP 4*, *6-1* and *6-2* may consistent with their potential multiple roles in chemoreception and contact chemoreception. Similar results were observed in *Bradysia odoriphaga, B. mori*, and *Nilaparvata lugens,* in which CSPs was expressed in antennae and the body (e.g. heads, wings, abdomen, and legs) (Gong et al., 2007; Waris et al., 2018; Younas et al., 2018; Zhang et al., 2021), suggesting the potential roles in contact chemoreception. Because *EonuCSP 4*, *6-1* and *6-2* expression were also found in heads, antennae and legs, we speculated that these 3 CSPs function in chemosensation.

The predicted 3D structure of the 3 CSPs exhibited conserved structural features, including six α-helices that form the hydrophobic ligand-biding pocket (Lartigue et al., 2002; Zhang et al., 2021). To further infer the potential roles of *EonuCSP 4*, *6-1* and *6-2*, the fluorescence competitive binding assay were performed. Of the 15 tested compounds, only 3 compounds showed relatively high binding affinities (*K*_i_ < 20 μM) for *EonuCSP4* (binding to farnesene and ocimene) and *EonuCSP6-1* (benzaldehyde), whereas EonuCSP6-2 showed weak binding to all the tested compounds (*K*_i_ > 20 μM). These results indicated that *EonuCSP4* and *EonuCSP6-1* might have the appropriate binding sites for the compounds, which involved in chemoreception of *E. onukii*. Behavioral responses also showed that adult *E. onukii* exhibited a significant preference for benzaldehyde, which was confirmed by previous studies (Cai et al., 2017). Benzaldehyde is a prominent scent compound which greatly contribute to the fruity, floral smells of flowers, plants, and fruits. Also, benzaldehyde has a typical almond-like odor, which is one of the key volatile components in all types of tea, especially green tea, black tea, and oolong tea (Yang et al., 2013; Wang X. et al., 2019). Thus, these findings indicated that *EonuCSP6-1* is a chemoreception protein, which may be involved in attraction of *E. onukii* adults to host. Behavioral responses also indicated that adult *E. onukii* exhibited a significant preference for Cis-3-Hexenyl n-valerate. However, we did not identified the potential CSPs that involved in binding this compound.

In summary, we report the expressions and ligand binding capabilities of 3 CSP of *E. onukii*, proving the potential olfactory roles of CSPs in host-location behavior of tea leafhopper. Totally, three volatiles exhibited strong binding abilities for CSPs in *E. onukii*, and one of them were able to elicit behavioral responses. These results provide clues into the mechanism of olfactory recognition of *E. onukii*. Besides, this study indicate that the CSPs in *E. onukii* are involved into the functions of host plant volatiles perception, and can be used as a molecular target for screening behaviorally active compounds for alternative control strategies.

## Data Availability

The datasets presented in this study can be found in online repositories. Coding sequences of EonuCSP4, 6-1, and 6-2 are deposited in GenBank with accession number MF509603.1, MF509616.1 and MF509617.1, respectively.

## Supplementary material

Supplementary Figure S1 PCR amplification of 3 EonuCSPs. Line 1 represents another EonuCSP lacking two α-helices, which was exclude from further analysis.

Supplementary Figure S2 Chromosomal distribution of *EonuCSP4, 6-1*, and *6-2*.</b> All these three genes were located on chromosome 1 of *E. onukii.*

Supplementary Figure S3 PROCHECK results from the predicted 3D model of *EonuCSP4, 6-1*, and *6-2*. Ramachandran plot showed that > 85% of the residues were in preferred regions, all the residues (100%) were in the allowed region.
