# Supplementary material for: Involvement of three chemosensory proteins in perception of host plant volatiles in the tea green leafhopper, Empoasca onukii
Source: Front Physiol. 2023 Jan 4;13:1068543. doi: 10.3389/fphys.2022.1068543 (PMC9845707; doi:10.3389/fphys.2022.1068543)
Supplement: Supplementary file 1 [file Table1.DOCX]

Table S1. Primers were used in qPCR and designed to amplify the coding regions.

| Gene | Forward (5'-3') | Reverse (5'-3') |
| --- | --- | --- |
| Primers used in qRT-PCR | | |
| EounCSP4 | CACTCAGAAGCAGAAGGAGAAG | CTGGTGTAAGTGCCGTCAG |
| EounCSP 6-1 | AGCAGTACACCACCAAGTTC | ACATCCTCCCTCGTCCAT |
| EounCSP6-2 | GACTGCTGAAGACTTACCTCAA | TTCTCCGTACACTTGGAACAC |
| β-actin | AGCGTGGTTACTCTTTCA | GCAACTCGTAGGACTTCT |
| Primers used for amplifying the coding regions | | |
| EounCSP4 | ATGAAGGTGGCAGTGTGTTTTG | TTACTGTTTGCCTTTCTTGAACTTGCTGGTG |
| EounCSP 6-1 | ATGATGAGCCTGAGAGTCCTCC | CTATTCGACCTTGACACCCTCC |
| EounCSP6-2 | ATGTCGACGTGTACTCTGGTGTTG | TCAGACCTTGACACCCTCTCGC |
| Primer sequences for prokaryotic expression | | |
| EounCSP4 | TGCTCGAGTGCGGCCGCTGATTCCAGTACGTACTCCACAG | GACGACAAGGCCATGTTACTGTTTGCCTTTCTTGAACTTGCTGG |
| EounCSP 6-1 | TGCTCGAGTGCGGCCAAGGCTGAAGACAAGCAGTAC | GACGACAAGGCCATGCTATTCGACCTTGACACCCTCCT |
| EounCSP6-2 | TGCTCGAGTGCGGCCGGGGATGAAAGCTACACCAGCCG | GACGACAAGGCCATGCTATTCGACCTTGACACCCTCCT |
