# Supplementary figures and images for: Involvement of three chemosensory proteins in perception of host plant volatiles in the tea green leafhopper, Empoasca onukii
Source: Front Physiol. 2023 Jan 4;13:1068543. doi: 10.3389/fphys.2022.1068543 (PMC9845707; doi:10.3389/fphys.2022.1068543)

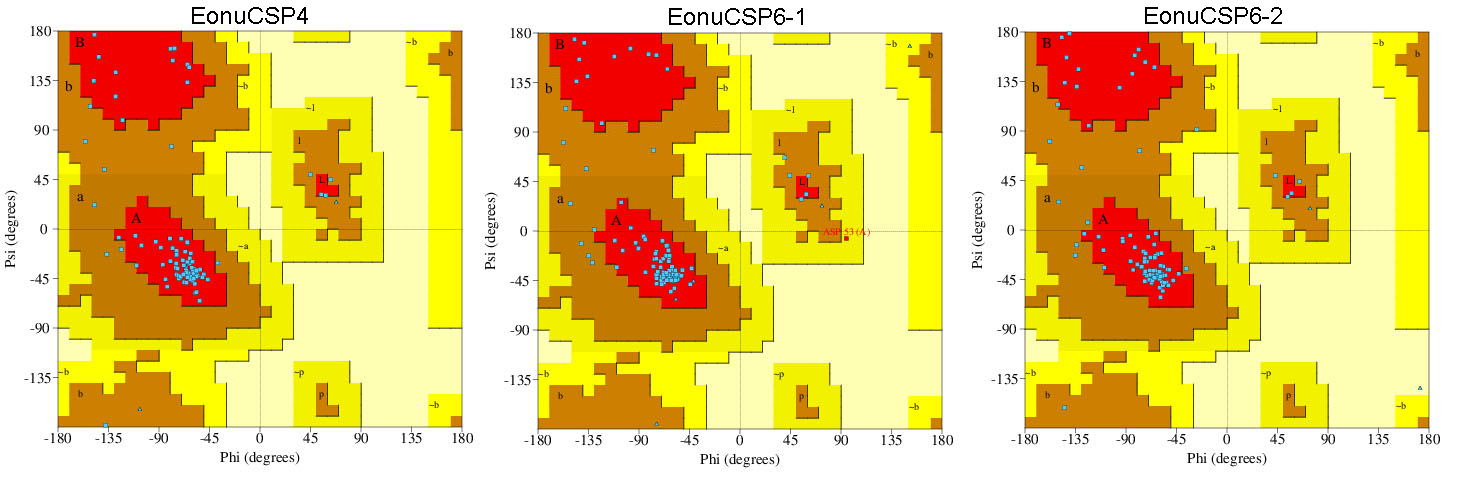

Supplement: Supplementary file 2 [file Image3.TIF]

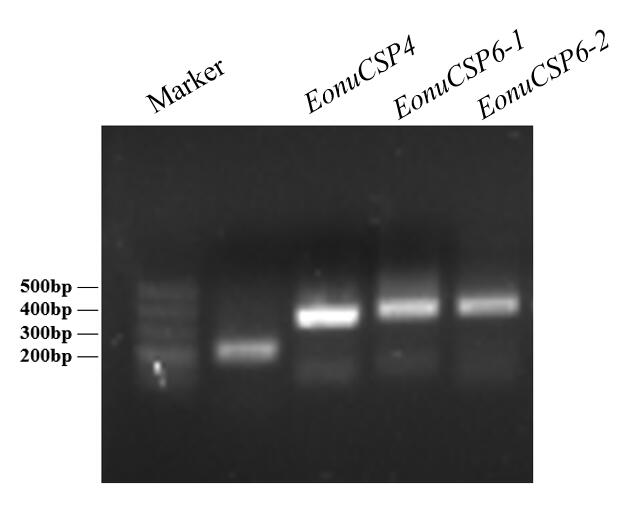

Supplement: Supplementary file 3 [file Image1.JPEG]

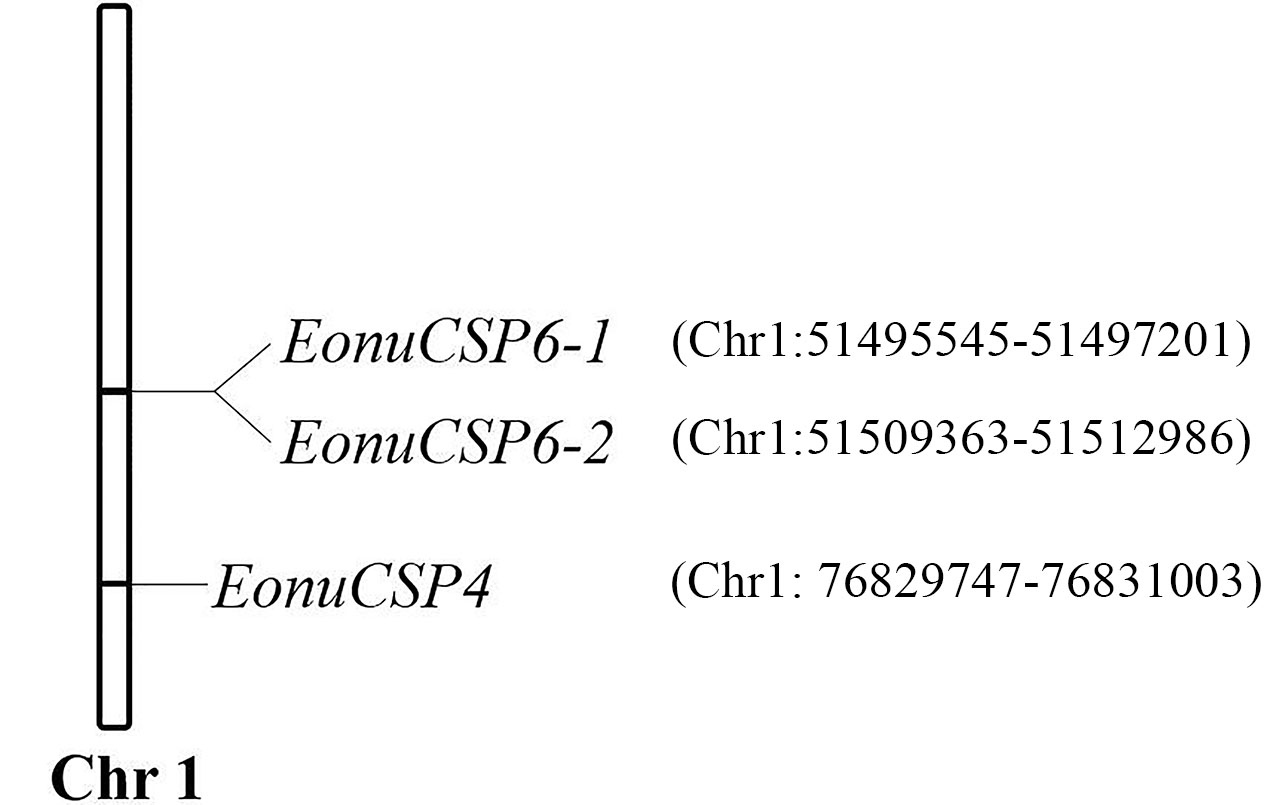

Supplement: Supplementary file 4 [file Image2.JPEG]
