# Supplementary material for: Involvement of three chemosensory proteins in perception of host plant volatiles in the tea green leafhopper, Empoasca onukii
Source: Front Physiol. 2023 Jan 4;13:1068543. doi: 10.3389/fphys.2022.1068543 (PMC9845707; doi:10.3389/fphys.2022.1068543)
Supplement: Supplementary file 5 [file Table2.DOCX]

Table S2. Information for protein 3D modeling on SWISS MODEL

| Protein | Modeling strategies | Template | Seq Identity % | Coverage % | GMQE | QMEAN |
| --- | --- | --- | --- | --- | --- | --- |
| CSP4 | SWISS MODEL | 2gvs.1.A | 47 | 81 | 0.64 | -0.85 |
| CSP6-1 | SWISS MODEL | 2gvs.1.A | 46 | 79 | 0.66 | -0.28 |
| CSP6-2 | SWISS MODEL | 2gvs.1.A | 52 | 33 | 0.64 | -0.22 |
