# Supplementary material for: Involvement of three chemosensory proteins in perception of host plant volatiles in the tea green leafhopper, Empoasca onukii
Source: Front Physiol. 2023 Jan 4;13:1068543. doi: 10.3389/fphys.2022.1068543 (PMC9845707; doi:10.3389/fphys.2022.1068543)
Supplement: Supplementary file 6 [file Table3.DOCX]

Table S3. Sequences information of the 3 CSP in *E. onukii.*

| Gene | ORF length (bp) | Signal peptides (bp) | Molecular Weight (KDa) | Isoelectric point (IP) |
| --- | --- | --- | --- | --- |
| *EonuCSP4* | 372 | 54 | 13.61 | 9.08 |
| *EonuCSP6-1* | 396 | 54 | 15.06 | 8.21 |
| *EonuCSP6-2* | 399 | 54 | 15.12 | 9.34 |
